# *Culicoides insignis* Lutz and *Culicoides stellifer* (Coquillett) (Diptera: Ceratopogonidae) are more active at canopy height than ground level throughout the night in Florida

**DOI:** 10.1093/jme/tjag002

**Published:** 2026-01-30

**Authors:** Vilma M Cooper, Simon S Riley, Terry Debriere, Samantha M Wisely, Juan M Campos-Krauer, Eva A Buckner, Nathan D Burkett-Cadena

**Affiliations:** Florida Medical Entomology Laboratory, Department of Entomology and Nematology, Institute of Food and Agricultural Sciences, University of Florida, Vero Beach, FL, USA; Statistical Consulting Unit, Institute of Food and Agricultural Sciences, University of Florida, Gainesville, FL, USA; Grow Green Technologies LLC, Jacksonville, FL, USA; Wildlife Ecology and Conservation Department, University of Florida, Gainesville, FL, USA; Wildlife Ecology and Conservation Department, University of Florida, Gainesville, FL, USA; Department of Large Animal Clinical Sciences, University of Florida, Gainesville, FL, USA; Florida Medical Entomology Laboratory, Department of Entomology and Nematology, Institute of Food and Agricultural Sciences, University of Florida, Vero Beach, FL, USA; Florida Medical Entomology Laboratory, Department of Entomology and Nematology, Institute of Food and Agricultural Sciences, University of Florida, Vero Beach, FL, USA

**Keywords:** circadian rhythms, vector control, adulticide timing, *Culicoides insignis*, *Culicoides stellifer*

## Abstract

*Culicoides* biting midges (Diptera: Ceratopogonidae) are important, yet understudied vectors of animal and human pathogens. Many biting midge species reach significantly higher densities at tree canopy height (10-20 m), compared to ground level (1 m), which may compromise the effectiveness of space spray ground adulticiding. Timing adulticide applications to coincide with the hour(s) of greatest midge density at ground level could ameliorate this issue. However, the circadian activity patterns for most vector species remain poorly understood, especially in regard to vertical movement (between ground and canopy). This study investigated the circadian flight patterns and vertical stratification of two vector species (*Culicoides insignis* Lutz and *Culicoides stellifer* [Coquillett]) using a novel hourly sampling light trap operated at ground (≈1 m) and canopy (≈11 m) heights from 1700 h to 0800 h on two Florida deer farms. Both *C. insignis* and *C. stellifer* exhibited strong vertical stratification, with significantly more midges collected in the canopy than at ground level, up to 7.4-fold and 11.5-fold differences, respectively. Peak flight activity was not crepuscular, but occurred between 0028 h and 0103 h for *C. insignis*, and between 2240 h and 0234 h for *C. stellifer*, varying somewhat by location and height. The circadian and vertical activity patterns observed here contrast with assumptions of crepuscular activity in *Culicoides* spp. This suggests that the current timing of ultra-low volume insecticide applications on deer farms (typically dusk) is not aligned with peak *Culicoides* activity (2200 h–0200 h), reducing efficacy of control measures. To optimize *Culicoides* control outcomes, adulticides should be applied when *Culicoides* activity peaks.

## Introduction

*Culicoides* biting midges (Diptera: Ceratopogonidae) are important yet understudied vectors of animal and human pathogens. Two key aspects of adult *Culicoides* behavior, circadian activity patterns and vertical stratification, can vary widely among species and environments, with direct implications for surveillance and control. As a group, *Culicoides* are typically described as crepuscular, exhibiting peak host-seeking activity at dawn and dusk (Viennet et al. 2012, Meiswinkel and Elbers 2016, Lakew et al. 2021, Zhang et al. 2024). However, their 24-h activity patterns remain poorly characterized due to limited rigorous sampling (Barnard and Jones 1980, Lillie et al. 1987). *Culicoides* species that transmit pathogens to ungulates bite their hosts at ground level, yet a growing body of literature has demonstrated that these same vector species ascend to the tree canopies, in large numbers, for unknown reasons (McGregor Bethany et al. 2018, Kessinger et al. 2025).

Exceptions to the crepuscular activity paradigm of *Culicoides* are well documented. For example, *Culicoides paraensis* Goeldi, a vector of Oropouche virus, displays diurnal peaks between 1600 h and 1800 h during the rainy season in Brazil (Feitoza et al. 2023). In Florida, nuisance species such as *Culicoides furens* (Poey) and *Culicoides mississippiensis* Hoffman are primarily active during evening twilight but may shift toward daytime activity in cooler conditions (Bidlingmayer 1961, Lillie et al. 1987). In temperate North America, *Culicoides sonorensis* Wirth and Jones, a confirmed vector of epizootic hemorrhagic disease virus (EHDV) and bluetongue virus (BTV) peaks at dusk and dawn in summer, shifting to earlier evening activity during cooler months (Nelson and ­Bellamy 1971, Barnard and Jones 1980). Zhang et al. (2024) also observed this crepuscular pattern in *C. sonorensis*, with later activity peaks on warmer nights, suggesting temperature-dependent plasticity in host-seeking behavior. In Europe, *Culicoides obsoletus* (Meigen), a vector of BTV and Schmallenberg virus, exhibits predominantly nocturnal activity, with peaks between 2200 h and 0400 h (Meiswinkel and Elbers 2016). These examples highlight the need for species and region-specific characterization of *Culicoides* circadian activity.

*Culicoides* species often exhibit strong vertical stratification, with some species significantly more abundant in tree canopies than near the ground. For example, *Culicoides stellifer* (Coquillett) has been captured in 5-fold greater numbers at 12 m than at 6 m in New Jersey light traps (Henry and Adkins 1975). At a Florida deer farm, *C. stellifer* was captured in 11-fold greater numbers in the forest canopy (≈9 m) compared to ground level (≈1 m) using modified CDC light traps (McGregor Bethany et al. 2018). Similarly, *Culicoides insignis* Lutz, a confirmed vector of BTV in South America, the Caribbean, and the Southeastern United States (McGregor Bethany et al. 2018, Kessinger et al. 2025) was up to six times more abundant at 9 m than at ground level in the same study (McGregor Bethany et al. 2018). *Culicoides venustus* Hoffman also showed a tendency toward greater canopy abundance, with up to 14 times more individuals captured at 9 m. Vertical stratification is suspected to be influenced by host availability (Swanson et al. 2012), host size and vertical positioning (Tanner 1971), or environmental factors such as light intensity (Snow 1955), although the precise drivers remain uncertain (Pfannenstiel et al. 2015).

Despite the recognized importance of both temporal and vertical aspects in *Culicoides* ecology, these factors have rarely been studied together. One notable exception is Snow (1955), who demonstrated that the abundance of *Culicoides* spp. at different heights changes throughout the day and night. For example, *C. paraensis* was observed to move toward the canopy during the day, with diurnal feeding primarily occurring at ground level and very few individuals feeding in the forest canopy. Understanding how time of day interacts with height is critical for practical applications of control measures. For instance, ultra-low volume (ULV) adulticide sprays, typically applied from ground-based vehicles, are most effective when timed to coincide with peak vector activity at the relevant heights (Kalmouni et al. 2024).

Understanding circadian activity and vertical stratification of *Culicoides* is particularly important in Florida deer farms, where outbreaks of EHDV and BTV cause severe morbidity and mortality in white-tailed deer (Cottingham et al. 2021). Two species, *C. insignis* and *C. stellifer*, are prevalent on these farms and are critical targets for vector surveillance and control. Limited available data suggests that *C. insignis* is crepuscular, with activity after sunset and in the early morning (Corrêa et al. 2007), but its full circadian patterns remain insufficiently explored. For *C. stellifer*, circadian activity patterns are essentially unstudied, especially with respect to vertical movements. If vector species remain aloft (above 10 m) during hours when space spray ground adulticiding is typically conducted (dusk and shortly thereafter), then the efficacy of adulticide applications may be severely compromised. For example, Kim et al. (2022) showed that height significantly impacted mosquito mortality in caged semi-field trials with a malathion space spray ground adulticide application, observing 100% mortality in mosquitoes at 1 m and 4 m heights, but 0–8% mortality in mosquitoes at 7 m height. Therefore, vertical and circadian activity patterns of *Culicoides* could result in apparent behavioral avoidance of contact with adulticides. Permethrin-based space spray ground adulticiding is the most widely used *Culicoides* control method on Florida deer farms (Harmon et al. 2020, Cooper et al. 2025b). These applications have been shown to achieve 100% mortality against local EHDV and BTV vector species in caged semifield trials (Cooper et al. 2025a). However, adulticide application times vary across deer farms, with 44% applying at dusk, 16% at dawn, 16% during the day, and 20% at opportunistic times, highlighting a lack of evidence-based guidelines for targeting these vectors (Cooper et al. 2025b). Given that space spray adulticide efficacy relies on droplets hitting flying *Culicoides*, aligning spray timings with peak activity at ground level could substantially improve control outcomes.

The objective of this study was to characterize the circadian flight activity and vertical stratification of *C. insignis* and *C. stellifer* on Florida deer farms during the EHDV and BTV transmission season (McGregor Bethany et al. 2019b). Using a novel hourly sampling light trap operated at ground (≈1 m) and canopy (≈11 m) heights from 1700 h to 0800 h, we sought to identify periods and heights of maximum flight activity. These data can inform optimized timing and placement of ULV insecticide applications, thereby improving the efficacy of vector control programs.

## Materials and Methods

### Study Locations

The study was conducted at two privately-owned deer farms in Florida. One farm was located in Gadsden County (northern Florida), and encompassed 200 ha, including 10 high-fenced breeding pens (8.5 ha) housing white-tailed deer. Pens were surrounded by 191.5 ha of a free-range preserve with diverse native (white-tailed deer) and exotic (nilgai, axis, and black buck) ungulate species. Putative EHDV vector, *C. stellifer* was the predominant biting midge species during late summer and fall (HD season) (McGregor Bethany et al. 2018, 2019a, 2019b). The second farm was located in southern Florida (Martin County). The area featured seasonally flooded wet prairies adjacent to cattle pastures. The property housed only white-tailed deer in six pens. Previous studies indicate that *C. insignis*, a main BTV vector in Florida, was the dominant biting midge species at this site (Sloyer et al. 2023).

### Day Versus Night Activity

**Fig. 1.**
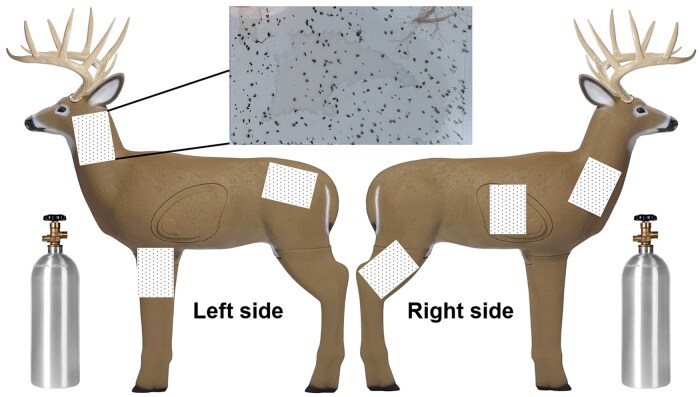
Deer simulator used to estimate *Culicoides insignis* Lutz landings on deer. Each deer 3D shooting target had three sticky cards affixed to both sides of their neck, back, and legs and were baited with compressed carbon dioxide delivered through a gas regulator (Aquarzon, Australia) set to 75 kgf/cm^2^.

A preliminary experiment was conducted at the Martin County farm from October 1 to 4 to test the assumption that *C. insignis* was mainly active at night, hence, our focus on hourly sampling during the night for this species. Because light traps would likely be ineffective during the daytime we used “deer simulators” designed to mimic an adult white-tailed deer (Fig. 1), which served as a visual and olfactory lure for host-seeking *Culicoides* that are active either day or night. Each simulator consisted of a 1.5 m tall deer-shaped shooting target made of foam and plastic (FeraDyne Outdoors, Fond Du Lac, WI, USA; Fig. 1). Simulators were baited with compressed carbon dioxide ­delivered through a gas regulator (Aquarzon, Australia) set to 75 kgf/cm^2^ (Cooper et al. 2025). Six sticky cards (15 × 7.9 cm; Biogents USA, Cary, NC, USA) were placed on both sides of the deer simulator’s neck, back, and legs to capture landing insects (Fig. 1), these sites selected based on preliminary observations of preferred landing areas (Cooper, unpublished data). Cards were collected and replaced daily, with separate 12-h sampling intervals for daytime (0600 h to 1800 h) and nighttime (1800 h to 0600 h). *Culicoides* were identified to species using keys in Blanton and Wirth (1979) and Blosser et al. (2024).

### Programmable Rotating Trap for Hourly Sampling

**Fig. 2.**
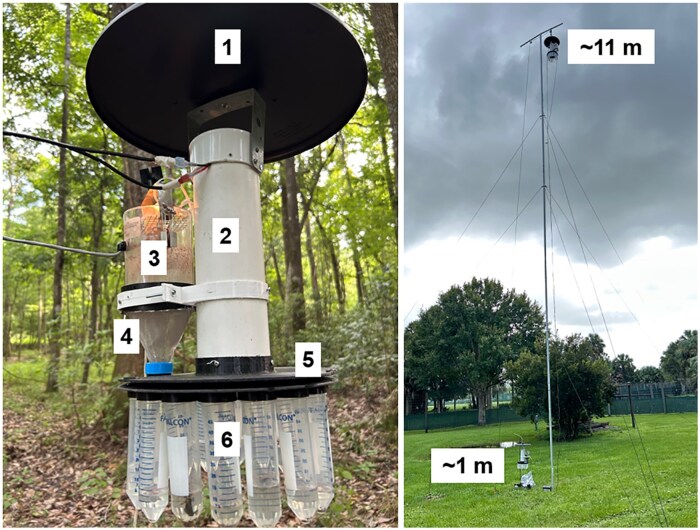
Novel programmable rotating trap used to collect *Culicoides* biting midges over time at ground and canopy heights. Left: Schematic of 16-h collection trap: (1) rain shield, (2) PVC tube containing printed circuit board and motor powered by a 12 V battery, (3) CDC light trap baited with an incandescent light bulb powered with a 6 V battery, (4) stainless-steel mesh funnel to deliver insects into collection tubes, (5) rotating plate with 16 openings, (6) 50 ml Falcon tubes. Right: Placement of trap at ground (≈1 m) and canopy (≈11 m) heights.

A novel programmable rotating trap was designed and built (GrowGreen Technologies LLC, Jacksonville, FL, USA) to collect insects in 16 separate samples, over time (Fig. 2). The trap employed a rotating disc consisting of a 16-hole turntable powered by a stepper motor (NEMA 17 59 Ncm 2A), with a real-time clock (PFC8523), and microprocessor (ESP32-C3) for programming the disc to rotate at predetermined times (Fig. 2). The turntable held 16, 50 ml Falcon tubes with isopropyl alcohol (70%) to collect a maximum of 16 samples throughout a 16-h period. A Centers for Disease Control and Prevention (CDC) miniature light trap baited with one incandescent light bulb was secured to the central housing with 3D-printed hangers and served as the suction device, blowing insects attracted to the light through a screen mesh funnel, into alcohol within the tube. The trap was programmed daily using the ESP32 BLE Terminal smartphone application (App Store, Apple) to set operational times.

### Circadian and Vertical Activity of *C. insignis* and *C. stellifer*

*Culicoides* were trapped using the novel programmable rotating trap to characterize the circadian activity and vertical stratification of *C. insignis* in Martin County and *C. stellifer* in Gadsden County. In 2023, traps were deployed at ground (≈1 m) and canopy (≈11 m) levels at both farms. Although *Culicoides* have been observed flying as high as 1.5 km (Glick 1939), our study focused on heights below 12 m, which are previously shown to be ecologically relevant for *C. stellifer* and *C. insignis* (McGregor Bethany et al. 2018). Ground-level traps were suspended from a shepherd’s hook at ≈1 m above the ground, while canopy traps were positioned ≈11 m above the ground using a pole constructed from six 1.8 m × 4.44 cm aluminum swaged button handles (Anvil, Home Depot, Atlanta, GA, USA) and secured with 0.32 cm black Dacron guy ropes (Max-Gain Systems, Marietta, GA, USA) tied to three ground anchors (Fig. 2). A pulley system was integrated at the top of the pole, allowing canopy traps to be raised and lowered easily for sample retrieval without dismantling the structure.

At the Gadsden County farm, sampling occurred in the preserve area, where deer were free to roam, with traps located at least 500 m from the deer pens. Two contrasting sites were selected: a “closed canopy” site dominated by hardwoods and heavy shade, and an “open canopy” site in a pine-dominated area with sparse cover, providing little to no shading. At the Martin County farm, two trap sites were established: the “driveway,” located along an open, partially shaded path adjacent to deer pens, and the “house,” situated between a residential structures and deer pens. These sites were about 100 m apart and consisted of similar habitat structure; therefore, differences in *Culicoides* activity were not anticipated. One trap per height (ground and canopy) were placed at the two sites within each farm. Sampling occurred hourly from approximately 1 h before sunset until 1 h after sunrise (1700 h to 0800 h) for five consecutive nights every four weeks from June to November, totaling 40 sampling nights per farm. Specimens were identified to species based on external morphology using dichotomous keys and species descriptions from Blanton and Wirth (1979) and Blosser et al. (2024).

### Parity and Circadian Flight Activity of *C. insignis* at Ground Level

A separate experiment was conducted to assess whether the nocturnal activity of *C. insignis* varied by parity status. In 2024, two ground-level traps were operated at the Martin County farm targeting *C. insignis* for 10 sampling nights (20 sampling nights total). Traps were run for 13 h each night, from 1800 h to 0600 h. Female *C. insignis* were examined to determine their parity status (nulliparous, parous, or bloodfed) as described by Dyce (1969).

### Data Analysis

A Wilcoxon signed-rank test was performed to compare *C. insignis* counts collected from deer simulators at daytime versus nighttime across eight replications. Data on the circadian and vertical activity of *C. insignis* and *C. stellifer* were analyzed using R version 4.4.0 (R Core Team 2024) and the tidyerse suite of packages (Wickham et al. 2019). Sampling nights with fewer than 10 *C. insignis* or fewer than four *C. stellifer* were excluded from analysis because they provide insufficient information for estimating temporal patterns and disproportionately inflate model uncertainty. Generalized linear mixed models (Gbur et al. 2012) with negative binomial distribution and log link function were fit to the midge count data—with data from each species analyzed separately—using the glmmTMB package (Brooks et al. 2017). Akaike Information Criterion (AICc) was employed, via the performance package (Lüdecke et al. 2025), to select the best from among a set of candidate models which differed in the fixed effects employed to characterize the temporal trend in midge catch rates. AICc was further employed to determine whether random effects should include only a random intercept for each day, or also random slopes or trends, and to verify that alternative response distributions did not improve model fit. Finally, model assumptions assessed using visual inspection of quantile residual diagnostic plots using the DHARMa package (Hartig and Lohse 2022).

Having identified the best fitting model and having validated that there was no evidence that the models’ assumptions were violated, a Wald Chi-square test was performed on the fixed effects using the car package (Fox et al. 2023), and marginal mean trends for each combination of trap height and location were calculated using the marginaleffects package (Lüdecke et al. 2025). Finally, the delta method was employed to derive from the coefficients of the fitted model estimates and confidence intervals of both the time at which midge catch peaked, and the maximum hourly midge catch rate for each combination of trap height and location (Bolker 2008).

A separate GLMM was used to analyze the average counts of parous *C. insignis* collected at ground level over 13 h periods in 2024. A negative binomial distribution was applied to account for over dispersion, with time as a fixed effect. Post hoc comparisons were performed using estimated marginal means to evaluate differences in parous activity across sampling hours. A linear regression analysis was performed to explore the relationship between the counts of nulliparous and parous *C. insignis* collected at ground level.

## Results

### Day Versus Night Activity

**Fig. 3.**
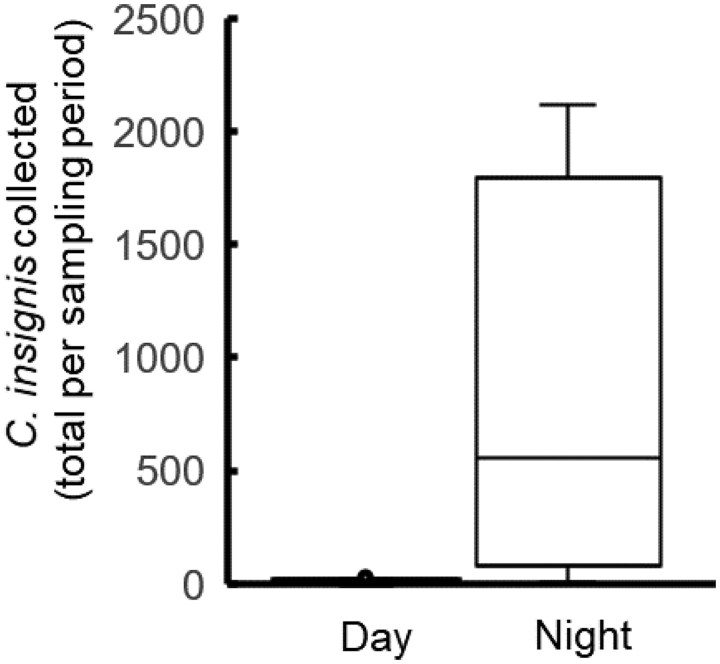
Daytime vs nighttime activity of *Culicoides insignis* Lutz females at a deer farm in Martin County, Florida (1–4 October 2024). Boxplot shows total females collected per 12-h sampling period (day: 0600 h–1800 h and night: 1800 h–0600 h) using carbon dioxide-baited deer simulators with sticky cards.

The number of *C. insignis* females collected from deer simulators was 90 times greater during the nighttime (1800 h–0600 h) than daytime (0600 h–1800 h). An average of 829.75 females per deer simulator were collected during the nighttime, and 9.13 per deer simulator during the daytime (Fig. 3). A Wilcoxon signed-rank test confirmed significantly greater nocturnal activity (*P* = 0.0078), supporting our decision to conduct hourly sampling during nighttime to determine peak flight activity.

### Circadian and Vertical Activity of *C. insignis* and *C. stellifer*

**Fig. 4.**
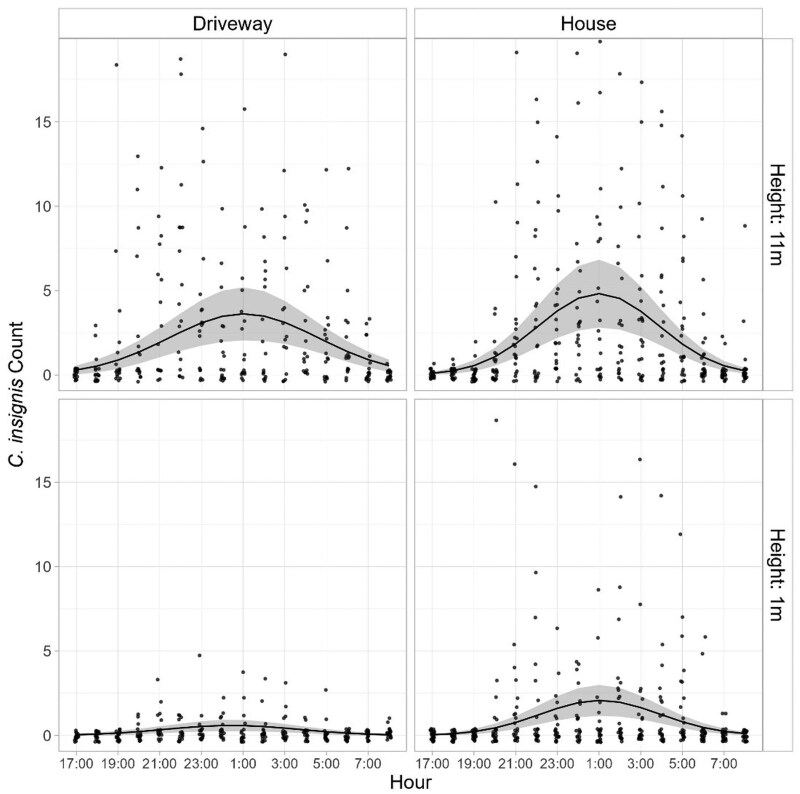
Circadian flight activity of *Culicoides insignis* Lutz at ground (1 m) and canopy (11 m) heights on a deer farm in Martin County, Florida. Estimated mean counts (solid lines) with 95% confidence intervals (shaded areas) are shown for two sampling locations, based on hourly light trap collections from 1700 h to 0800 h (40 sampling nights).

**Fig. 5.**
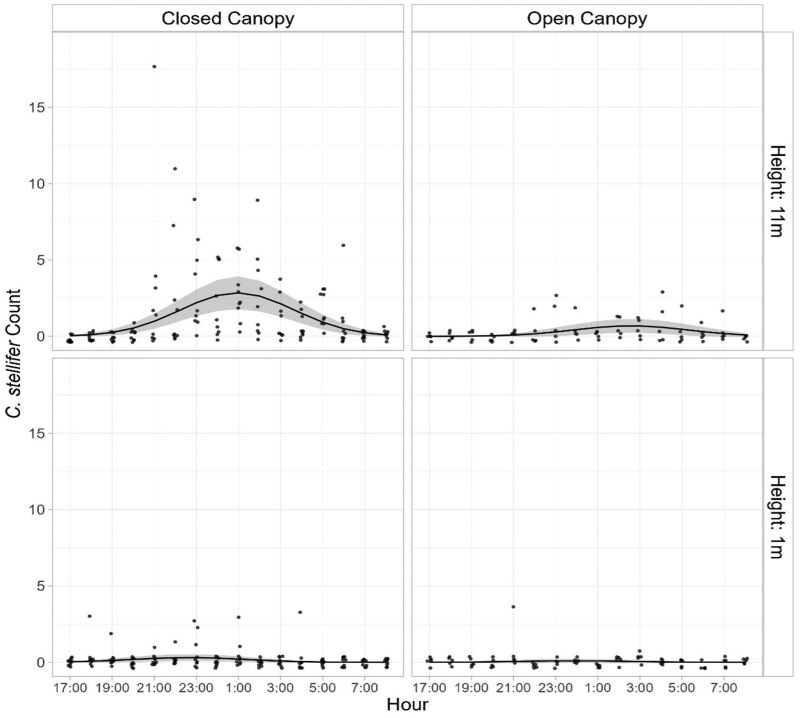
Circadian flight activity of *Culicoides stellifer* (Coquillett) at ground (1 m) and canopy (11 m) heights on a deer farm in Gadsden County, Florida. Estimated mean counts (solid lines) with 95% confidence intervals (shaded areas) are shown for two sampling locations, based on hourly light trap collections from 1700 h to 0800 h (40 sampling nights).

Flight activity patterns of *C. insignis* and *C. stellifer* were strongly nocturnal and exhibited clear vertical stratification, with peak activity for both species occurring between approximately 2200 h and 0400 h. Activity was significantly influenced by both height and site (*P* < 0.0001), with greater captures in the canopy (≈11 m) than at ground level (≈1 m) at all sites (Figs 4 and 5). The extent of vertical stratification varied by species and site.

For *C. insignis* at the Martin County farm in southern Florida, vertical stratification was especially evident at the “driveway” site, where nightly catches of *C. insignis* were 7.4 times greater at the canopy level than at ground level (95% CI: 4.6–11.83, *P* < 0.0001; Fig. 4). A similar pattern was observed at the “house” site, with 2.4 times more *C. insignis* collected at the canopy level (1.7–3.3, *P* < 0.0001). For *C. stellifer* at the Gadsden County farm in northern Florida, canopy level catches were 5.6 times greater than ground level at the “open canopy” site (95% CI: 1.2–26.7, *P* < 0.0001) and 11.5 times greater at the “closed canopy” site (Fig. 5).

Flight activity of both species was strictly nocturnal, but peak timing differed (Figs 4 and 5). *Culicoides insignis* reached peak activity between 0030 h and 0100 h across sites and heights (Fig. 4). At ground level, peak activity occurred at 0103 h (95% CI: 0025 h–0141 h) at the house and 0028 h (2316 h–0139 h) at the driveway in Martin County in southern Florida (Fig. 4). During these peak times, the maximum hourly midge catches at ground level were 2.1 (1.3–3.2) at the house and 0.6 (0.3–1.1) at the driveway (Table 1). In the canopy, peak activity of *C. insignis* occurred at 0059 h (95% CI: 0029 h–0130 h) at the house and 0102 h (0012 h–0152 h) at the driveway (Fig. 4), with maximum hourly catches of 4.8 (3.2–7.3) at the house and 3.6 (2.4–5.6) in the driveway (Table 1).

**Table 1.** Peak activity times and maximum hourly catches of *Culicoides insignis* Lutz and *Culicoides stellifer* (Coquillett) at two heights and sites^a^

Species	Site	Height	Peak time	Max catch
***Culicoides insignis***	House	Canopy (11 m)	0059 h (0029 h–0130 h)	4.8 (3.2–7.3)
		Ground (1 m)	0103 h (0025 h–0141 h)	2.1 (1.3–3.2)
	Driveway	Canopy (11 m)	0102 h (0012 h–0152 h)	3.6 (2.4–5.6)
		Ground (1 m)	0028 h (2316 h–0139 h)	0.6 (0.3–1.1)
***Culicoides stellifer***	Closed Canopy	Canopy (11 m)	0057 h (0020 h–0134 h)	2.8 (2.0–4.2)
		Ground (1 m)	2240 h (2056 h–0023 h)	0.3 (0.2–0.6)
	Open Canopy	Canopy (11 m)	0234 h (0103 h–0406 h)	0.7 (0.4–1.3)
		Ground (1 m)	2356 h (2007 h–0345 h)	0.1 (0.0–0.4)

a Estimated time of peak activity (Peak Time) and the corresponding maximum number of midges caught per hour (Max Catch) of adults at ground (1 m) and canopy (11 m) heights on a deer farm in Martin County (*C. insignis*) and Gadsden County (*C. stellifer*), Florida. Values in parentheses indicate 95% confidence intervals.

*Culicoides stellifer* showed earlier and more variable peak activity, ranging from late evening to early morning depending on site and height (Fig. 5). At ground level, peak flight activity occurred at 2240 h (95% CI: 2056 h–0023 h) in the closed canopy and 2356 h (95% CI: 2007 h–0345 h) in the open canopy, with maximum hourly catches of 8.9 (8.3–9.5) and 7.9 (4.1–11.7) midges, respectively (Table 1). In the canopy, *C. stellifer* activity peaked at 0057 h (95% CI: 0020 h–0134 h) in the closed canopy and at 0234 h (0103 h–0406 h) in the open canopy, with maximum hourly catches of 2.8 (2.0–4.2) and 0.7 (0.4–1.3) midges, respectively (Table 1).

### Sex Ratios by Height and Time

**Fig. 6.**
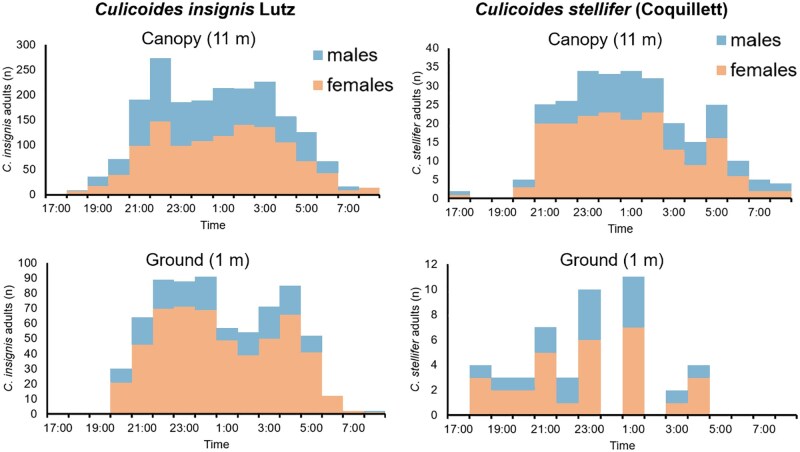
Total *Culicoides* spp. female to male ratio collected using a programmable rotating trap for hourly sampling during the season of EHDV and BTV transmission in 2023 in two Florida deer farms. Female to male ratio of *Culicoides insignis* Lutz in Martin County, FL (left) and *Culicoides stellifer* (Coquillett) in Gadsden County, FL (right) collected at ground (≈1 m) and canopy (≈11 m) heights between 1700 h and 0800 h.

Across both species, females consistently outnumbered males at all times and heights, though the magnitude of this bias varied (Fig. 6). For *C. insignis*, female predominance was particularly evident at ground level, with an average ratio of 3.2 females to every male, reaching a peak of 6:1 at 0100 h (Fig. 6, left). In the canopy, the ratio was lower but still female-skewed, averaging 1.7:1 throughout the night (Fig. 6, left). For *C. stellifer*, female predominance was less pronounced but still present, with average ratios of 1.65:1 at ground level and 2:1 in the canopy (Fig. 6, right). The greatest ratio at ground-level for *C. stellifer* was 3:1, occurring at both 1800 h and 0400 h.

### Parity and Circadian Flight Activity of *C. insignis* at Ground Level

**Fig. 7.**
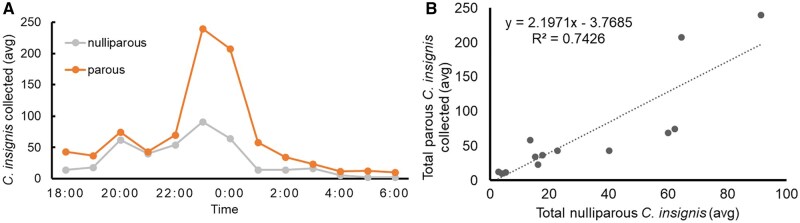
Activity and parity of female *Culicoides insignis* Lutz. A) Activity of nulliparous and parous females throughout the night at ground level. B) Scatterplot showing the relationship of the activity of parous and nulliparous females (*R*^2^ = 0.7426, *P* < 0.0001).

Parous *C. insignis* females were consistently the predominant physiological group across the 13 h-sampling periods. Parous *C. insignis* females were most active at 2300 h (*P* = 0.0319), followed by a notable decline at 0200 h (*P* = 0.0138; Fig. 7A). On average, 67 parous females were captured per sampling night, representing 60% of the total catch at ground level. Nulliparous individuals accounted for 28% (34 per night), males 11% (20 per night), and bloodfed females only 1% (two per night). A strong positive correlation was observed between parous and nulliparous female counts (*R*^2^ = 0.7426, *P* < 0.0001), suggesting shared drivers of activity (Fig. 7B). Two notable outliers at 2300 h and midnight reflected unusually high activity of parous individuals during these hours (Fig. 7B).

## Discussion

This study provides new insights into the temporal activity and vertical stratification of *C. insignis* and *C. stellifer*, two important vectors of hemorrhagic disease viruses in Florida. Collections using the deer simulators confirmed that *C. insignis* is predominantly nocturnal, validating our approach of nighttime sampling to identify peak vector activity. Although preliminary collections using the deer simulator could not be conducted in the Gadsden County farm, previous studies at this location have shown great abundance of this species throughout the night, and limited activity of *C. stellifer* during the day when aspirating midges directly from deer (McGregor Bethany et al. 2018, 2019b). Both species were primarily nocturnal but exhibited species-specific patterns in circadian flight activity and vertical stratification. These findings have direct implications for optimizing vector surveillance and control on Florida deer farms, particularly regarding the timing of space spray ground adulticiding. Both *C. insignis* and *C. stellifer* were significantly more abundant in canopy traps (11 m) than at ground level (1 m), with increases of up to 7.4-fold and 11.5-fold, respectively. These results corroborate previous findings by McGregor Bethany et al. (2018), who documented up to 11-fold and 6-fold greater abundance of *C. stellifer* and *C. insignis*, respectively, in canopy traps (≈9 m) compared to ground-level traps (≈1 m) at a deer farm in Gadsden County, Florida. The consistent presence of both species at elevated heights highlights the need to consider vertical stratification in vector control programs.

The ecological reasons for the presence of *C. insignis* and *C. stellifer* at canopy height remain unclear. McGregor Bethany et al. (2018) found that over 90% of bloodfed *C. stellifer* collected in the canopy had fed on ground-dwelling mammals, primarily deer, suggesting postfeeding vertical movement. Snow (1955) hypothesized that *Culicoides* may use tree canopies as resting sites. However, our observations of canopy activity at sites with limited or absent tree cover (e.g., house and driveway sites) in Martin County suggest that factors beyond resting behavior may influence vertical distribution. Further research is needed to identify the ecological or behavioral mechanisms driving this stratification.

The consistent presence of *C. insignis* and *C. stellifer* at canopy height suggests that conventional ground-based interventions may miss significant portions of active vector populations. The efficacy of space spray ground adulticiding has been shown to decrease at altitudes as low as four meters. Kim et al. (2022) found up to 98% survival in butterflies roosting at 7 m during ground ULV applications with malathion, suggesting that space spray ground adulticiding may not effectively reach *C. insignis* and *C. stellifer* at canopy heights. Aerial or drone-based adulticide applications, commonly used in agricultural and public health settings (Clayton and Sander 2002, Matthews 2021), may offer promising alternatives for targeting vectors in the canopy. However, their effectiveness against *Culicoides* spp. remains untested and warrants investigation. Additionally, space spray adulticide applications at higher altitudes could negatively impact pollinator populations (Kim et al. 2022).

We identified peak activity periods for *C. insignis* and *C. stellifer* during the hemorrhagic disease transmission season (late summer and fall). *Culicoides insignis* exhibited peak activity between midnight and 0103 h at both heights. Similarly, *C. stellifer* was most active between midnight and 0234 h in the canopy, but peaked earlier at ground level, as early as 2240 h. These nocturnal peaks contrast with other veterinary-relevant *Culicoides* species. For instance, *C. sonorensis* activity in California peaks shortly after sunset, with a smaller secondary peak at sunrise (Nelson and Bellamy 1971, Mullens 1995). Similarly, *Culicoides crepuscularis* Mall, a partially-incriminated vector of BTV and EHDV, is most active at dusk and dawn (Snow 1955), whereas another suspected vector, *Culicoides spinosus* Root and Hoffman, peaks in the afternoon and continues into twilight.

The activity patterns we observed in *C. insignis* and *C. stellifer* closely resemble those of *Culicoides obsoletus* (Meigen), the primary European vector of BTV, which is most active between 2200 h and 0400 h in early summer (May–June) in the eastern Netherlands (Meiswinkel and Elbers 2016). As seen in other *Culicoides*, circadian activity patterns may shift with seasonal changes. Viennet et al. (2012) found that *C. obsoletus* activity in western France peaked at sunset during summer (late June) but shifted to 1 h before sunset in spring (April–May) and fall (September–October) (Viennet et al. 2012). While our study took place during the EHDV and BTV transmission season, future research should explore how environmental factors such as temperature, humidity, and wind influence these patterns (Barnard and Jones 1980).

Understanding the activity patterns of infectious and host-seeking *Culicoides* is critical for effective vector control. Parous *C. insignis* at ground level were most active between 2300 h and 0200 h, aligning with their overall peak activity window. This is consistent with Mullens (1995), who reported that parous *C. sonorensis* were most active around midnight. Since parous midges are more likely to transmit EHDV and BTV (Dinh et al. 2021), focusing control efforts during their peak activity could improve effectiveness.

Female of both *C. insignis* and *C. stellifer* outnumbered males across all times and heights. In *C. insignis*, this predominance was most pronounced during the activity peak, likely reflecting the host-seeking behavior of biting females. In *C. stellifer*, the greatest female-to-male ratios (3:1) occurred at 1800 h and 0400 h, outside the overall activity peak (2240 h–2356 h). This mismatch may be due to the smaller sample sizes for *C. stellifer* in comparison to *C. insignis*. Alternatively, this discrepancy may indicate species-specific behavioral patterns, with *C. stellifer* engaging in secondary host-seeking periods at dusk and early morning, leading to elevated female ratios outside the main activity peak. Overall, males were more frequently collected in the canopy than at ground level, a pattern previously documented in *Culicoides*. For example, in the Baltic region of Europe, 91% of males were captured in traps placed at 13 m and 23 m, compared to only 9% at ground level (Bernotienė et al. 2024).

Our results suggest that current control strategies against EHDV and BTV vectors in Florida deer farms are poorly aligned with peak activity of *C. insignis* and *C. stellifer*. A 2023 survey of Florida deer farmers showed that space spray ground adulticiding was generally performed around dusk and dawn (Cooper et al. 2025b). The nocturnal patterns of *C. insignis* and *C. stellifer* observed in this study support shifting space spray ground adulticiding to between 2200 h and 0100 h to maximize efficacy. However, such timing may affect control of other pests and vectors, such as mosquitoes, and should be balanced accordingly.

Although our study provides detailed information on circadian and vertical activity, some limitations should be acknowledged. Activity data were pooled over the hemorrhagic disease season, which limits our ability to assess seasonal shifts in flight activity. We also focused on *C. insignis* in Martin County and *C. stellifer* in Gadsden County because each species predominates locally. As a result, our findings reflect activity patterns within these specific geographic and ecological contexts. Future studies should investigate how seasonal, environmental, and geographic conditions influence the behavior of *C. insignis* and *C. stellifer* to refine control strategies and deepen understanding of their ecology.
